# Supplementary material for: Single Cell Transcriptomic and Chromatin Profiles Suggest Layer Vb Is the Only Layer With Shared Excitatory Cell Types in the Medial and Lateral Entorhinal Cortex
Source: Front Neural Circuits. 2022 Jan 26;15:806154. doi: 10.3389/fncir.2021.806154 (PMC8826650; doi:10.3389/fncir.2021.806154)
Supplement: Supplementary file 14 [file Data_Sheet_1.ZIP › Exc_vs_Inh_Motifs/homerResults/motif4.similar.html]

motif4

## Information for motif4

C
G
T
A
A
G
T
C
C
G
T
A
G
A
T
C
T
A
G
C
C
A
G
T
A
C
G
T
A
T
C
G
  
Reverse Opposite:  

T
A
G
C
C
G
T
A
G
T
C
A
A
T
C
G
C
T
A
G
G
C
A
T
C
T
A
G
A
C
G
T
  

|  |  |
| --- | --- |
| p-value: | 1e-389 |
| log p-value: | -8.971e+02 |
| Information Content per bp: | 1.686 |
| Number of Target Sequences with motif | 24582.0 |
| Percentage of Target Sequences with motif | 58.27% |
| Number of Background Sequences with motif | 5218.7 |
| Percentage of Background Sequences with motif | 48.01% |
| Average Position of motif in Targets | 334.3 +/- 251.0bp |
| Average Position of motif in Background | 309.4 +/- 260.2bp |
| Strand Bias (log2 ratio + to - strand density) | 0.0 |
| Multiplicity (# of sites on avg that occur together) | 1.43 |
| Motif File: | file (matrix) reverse opposite |

### Similar de novo motifs found

|  |  |  |  |  |  |  |  |
| --- | --- | --- | --- | --- | --- | --- | --- |
| Rank | Match Score | Redundant Motif | P-value | log P-value | % of Targets | % of Background | Motif file |
| 1 | 0.633 | T A G C G T A C C T G A A G T C T A G C C A G T C G T A T C A G | 1e-197 | -454.483875 | 10.47% | 6.56% | motif file (matrix) |
| 2 | 0.719 | A C G T C G T A A G T C A C G T A C G T A G T C A C G T A C G T | 1e-149 | -344.884275 | 52.06% | 45.72% | motif file (matrix) |
